# The relationship between vocational college students’ liking of teachers and learning engagement: A moderated mediation model

**DOI:** 10.3389/fpsyg.2022.998806

**Published:** 2022-09-12

**Authors:** Lei Lu, Luyao Zhang, Longmei Wang

**Affiliations:** ^1^School of Business, Macau University of Science and Technology, Taipa, Macao SAR, China; ^2^Zhongshan Polytechnic, Zhongshan, China

**Keywords:** liking, psychological empowerment, perceived teacher support, learning engagement, moderated mediation model

## Abstract

To clarify the relationship between higher vocational students’ liking of teachers and their learning engagement, based on the theory of social exchange, 1,279 vocational students in the Yangtze River Delta and the Pearl River Delta in China are used as the research objects. From the perspective of students and teachers, SPSS and AMOS are used to conduct a two-stage linear regression analysis. The results show that (1) students’ liking of their teachers has a positive effect on learning engagement; (2) liking positively affects students’ psychological empowerment; (3) liking of teachers indirectly influences learning engagement through psychological empowerment; (4) teacher’s support positively moderates the indirect relationship between liking of teachers and learning engagement through psychological empowerment. This study attempts to provide practical guidance for college students to provide learning engagement.

## Introduction

Education revitalizes the country, and education has been the concern of the society and the country (Faqih and Jaradat, 2021; Shaturaev, 2021). As an important part of education, higher vocational education has attracted more and more attention from the government, enterprises, and students since The State Council of the People’s Republic of China puts forward the plan of vocational education reform and implementation in 2014 (Li and Pilz, 2021). The learning of higher vocational students is a multi-dimensional, complex, and dynamic process, and the degree of students’ engagement affects the output of learning results (Kimmel and Volet, 2010). Existing studies have found that learning engagement can not only improve students’ current academic performance (Kiuru et al., 2014), promote students’ academic achievement, and reduce the dropout rate (Klem and Connell, 2004), but also vertically predict students’ status of going to school or leaving school as well as their career and work (Alexander et al., 1997), which has a profound impact on student’s development and growth (Anderman and Patrick, 2012).

Learning engagement is a positive psychological state, which is mainly manifested by students’ involvement in their learning and classroom roles during learning, and their continuous and stable focus on completing their learning, which is characterized by vitality, dedication, and concentration (Schaufeli et al., 2002). Studies have confirmed that students with high learning engagement are enthusiastic and fully devoted to learning, which contributes to the learning gains of students (Skinner and Belmont, 1993). Therefore, it becomes more and more important to stimulate students’ engagement, especially when the internal and external environment of students’ competition becomes more and more complex.

To explore how to effectively stimulate the learning engagement of higher vocational students, existing scholars have found that gender, age, and grade of students have different relationships with learning engagement (Shiguang and Xinchun, 2011). Students’ family socioeconomic status also has different effects on their learning engagement (Qiao et al., 2013). Besides, the support of university teachers has a positive promoting effect on students’ learning engagement (Zhao and Qin, 2021). The degree of stimulating learning engagement is influenced by students’ factors, family and socioeconomic factors, and the teacher–student relationship. Most students’ learning engagement takes place in the classroom. In the relationship between students and teachers, teachers’ support for students affects students’ learning engagement (Liu et al., 2018). However, most of the existing studies take teacher–student one-way support as the cause of study learning engagement, whereas few take student–teachers’ liking as the cause variable of learning engagement. Therefore, the following questions are as follows: 1. Do students’ liking for their teachers affect their learning engagement? 2. Does teacher’s support promote the influence of the liking of teachers on their learning engagement?

The main idea of social exchange theory is to involve and maintain the relationship with others in the expectation of reward (Homans, 1958). In class, students interact with teachers and maintain a good relationship with teachers, which is conducive to more learning engagement. In an organizational enterprise, the theory points out that leaders and subordinates will maintain a sense of mutual benefit through a series of interactions, thus stimulating more input (Kong et al., 2017). This study is based on the social exchange theory, regarding the relationship between leaders and subordinates in organizational enterprises as that between teachers and students in universities. The degree of the devotion of students is determined jointly by themselves and teachers (Roth et al., 2007). Students’ liking for teachers and teachers’ support for students should be maintained in a state of mutual liking and appreciation, which is more conducive to stimulating more devotion to learning (McIntyre et al., 2021).

“Like” has emotional color, mainly manifested as a one-way positive cognition (Niven et al., 2012; Liu et al., 2020). Liking, as an emotional resource, affects the degree of students’ feedback on learning (Kong et al., 2017). Students’ liking for teachers shows their active learning in class and their preference for teachers (Hativa and Birenbaum, 2000). This kind of liking for teachers (from the bottom up) stimulates more learning input. While teachers’ liking for students is mainly manifested by their support for students, teacher’s support (from top to bottom) refers to teachers’ care and help for their students’ learning (Zhao and Qin, 2021). The more teachers support students, the more interest students have in their studies, and the more pleasure and sense of achievement they feel in the learning process, so they are more willing to invest more time, experience, emotion, and strategy in learning (Hong, 2012). This study explores from a binary perspective how students’ liking for teachers and teachers’ support for students affect students’ learning engagement.

**FIGURE 1:**
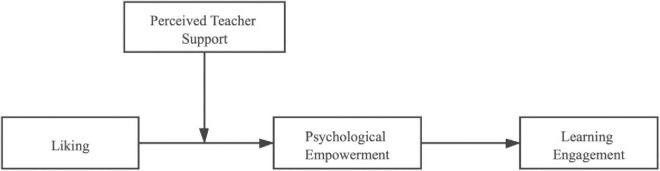
Research model diagram.

In summary, based on the social exchange theory, this study comprehends the relationship between students and teachers as a cooperative and mutually beneficial relationship between higher and lower levels. Students’ liking for teachers as bottom-up support reveals how liking affects students’ learning engagement in higher vocational colleges. Teachers’ support for students as top-down support reveals how teachers’ support affects students’ learning engagement in higher vocational colleges. This manuscript discusses the influence of liking and teacher’s support on learning engagement and how to interact through internal mechanisms from a dualistic perspective. Therefore, this study constructs a research model (Figure 1), which is mediated by psychological empowerment and moderated by perceived teacher’s support, to further clarify the internal mediation mechanism and boundary conditions between liking and students’ learning engagement in theory and explore the cause mechanism of learning engagement. It also provides some inspiration for college teachers to improve students’ learning engagement in educational practice.

## Research hypothesis

### Student–teacher’s liking and learning engagement

“Like” is a positive emotion of individuals, and students’ liking for their teachers will make students feel happy and thus have better performance. “Like” is a subjective judgment with emotion (Kong et al., 2017). The more students like their teachers, the more they seek enthusiasm, resources, and guidance for their learning. Students’ liking for their teachers will make them pay more attention to their teachers’ attention, and teachers’ attention to students is undoubtedly about their learning performance, vitality, and concentration. Once students have a positive liking for their teachers, students will show positive energy and concentration and become more actively involved in learning.

Learning engagement is a kind of positive psychological state (Yang et al., 2021). It is the behavior that students show enrichment, stability, and continuity in learning. It is the state in that students put themselves fully into learning, which can be divided into three dimensions: vitality, dedication, and concentration (Lee, 2014). Liking makes students feel happy, and positive emotions show better positive behavior. Students will always be energetic in learning, even in the face of learning difficulties that will actively adhere to, not be afraid. Because of the positive emotion of liking, students are fonder of learning and proud. Students will focus on the state of their study because they like to bring happiness, so when students are immersed in a happy learning atmosphere and focus on learning, it is difficult to get out of the study. To sum up, the positive liking emotion makes students feel happy, and students show energy, enthusiasm, and concentration in the learning process, which makes them more devoted to studying. Therefore, hypothesis 1 is proposed in this study:

*H1: Student–teacher’s liking positively influences student learning engagement.*

### Student–teacher’s liking and psychological empowerment

Psychological empowerment is a psychological state or a cognitive complex in which individuals experience being empowered (Spreitzer, 1995). It refers to the positive and meaningful experience that individuals obtain from the work of being motivated, satisfied, and fulfilled (Ahmed and Malik, 2019), and it is a kind of continuous motivation (Thomas and Velthouse, 1990). It is mainly manifested in four aspects: work meaning, self-efficacy, autonomy, and work influence. The meaning of students’ work is the cognition of students’ learning goals. Learning self-efficacy is the evaluation and cognition of students’ ability to complete work. Autonomy is the cognition of students’ learning control ability. The work influence of students is the cognition that students can influence themselves and others through learning (Zimmerman, 1995).

Student–teacher’s liking is a positive emotion that makes students feel happy and produces a state of mind that is motivated. Student–teacher’s liking makes students in a state of motivation. Students think that in a positive and valuable atmosphere, they can better understand the learning goals and significance, complete the assessment of learning ability, take the initiative to learn, and have a good impact on learning. Existing studies have found that when an individual is in liking, it is easy to establish a good exchange relationship (Kong et al., 2017), to reduce the objective obstacles to psychological empowerment, and more likely to trigger a positive attitude and maintain a high level of psychological empowerment. Therefore, hypothesis 2 is proposed in this study:

*H2: Student–teacher’s liking positively influences psychological empowerment.*

### The mediating role of psychological empowerment

As an individual’s inner positive emotion, liking can help students to better devote themselves to studying in a happy state (Abele-Brehm, 1992). Therefore, an in-depth analysis of the relationship between “emotion and behavior” from the perspective of student–teacher’s liking can effectively predict the level of student investment.

First, based on the social exchange theory, students are willing to devote more energy and time to learning when they like teachers, and teachers are willing to give students more resources and help when they feel the positive behavior of the students, so that students can get more learning resources, support, trust, and independence. Students perceive higher psychological empowerment. Therefore, they have more energy to study and show a higher level of learning involvement. Second, psychological empowerment can predict the level of engagement (Kong et al., 2017). The level of psychological empowerment will affect students’ attitudes toward learning and learning behavior (Fredrickson and Losada, 2005). Specifically, if students have high psychological empowerment, they will perceive more meaning and value affirmation of learning, then, they will have the belief to study harder, and they will make active efforts and actively invest in achieving their learning goals. Therefore, hypothesis 3 is proposed in this study:

*H3: Psychological empowerment plays a mediating role between student–teacher’s liking and learning engagement.*

### The moderating effect of perceived teacher’s support

Perceived teacher’s support refers to students’ feeling of encouragement and support from teachers (Babad, 1990), and obtaining valuable resource information, emotional identification, and pressure relief (Deci and Ryan, 1987), which is mainly manifested as teachers’ support for students in learning, ability, and emotion (Ou, 2005). When students have positive emotions of liking for teachers, students will have a satisfied and motivated happy state of mind and feel a high level of psychological empowerment. Based on the social exchange theory, students’ liking for teachers is a bottom-up positive emotion, while teachers’ support for students is a top-down encouragement. When these two are matched, first, students themselves will be happy and confident due to their liking for teachers. Second, the students will feel the support of the teachers, so that they can get more recognition and encouragement. In this way, students and teachers will establish close trust and respect, which will improve students’ psychological empowerment levels. When teachers provide support and help to students in learning, students will experience more pleasure and psychological empowerment (Abele-Brehm, 1992). Existing studies have found that in higher vocational colleges when students feel more encouragement and support from teachers in the learning process, they will show more interest, initiative, and pleasure (Zhao and Qin, 2021) and also invest more time, energy, and emotion in learning (Kong et al., 2017). Therefore, hypothesis 4 is proposed in this study:

*H4: Perceived teacher’s support has a positive moderating effect between student–teacher’s liking and psychological empowerment. In other words, the higher the students’ perceived teacher’s support, the higher the students’ psychological empowerment from the student–teacher’s liking.*

Combined with the relationship between hypotheses 3 and 4, it is concluded that psychological empowerment has a mediating role between student–teacher’s liking and learning engagement, and perceived teacher’s support has a positive moderating role between student–teacher’s liking and psychological empowerment. Therefore, this study further suggests that perceived teacher’s support positively moderates the mediating role of psychological empowerment between student–teacher’s liking and learning engagement. Specifically, students with a high level of perceived teacher’s support will be more cheerful and active and obtain higher psychological empowerment. Meanwhile, students with a high level of psychological empowerment will study more actively and harder and become more invested in learning. In conclusion, hypothesis 5 is proposed:

*H5: Perceived teacher’s support positively moderates the mediating effect of psychological empowerment between student–teacher’s liking and learning engagement. That is, the more students perceived teacher’s support, the relationship is stronger.*

## Research design and methodology

### Research model

Based on the social exchange model, this study explores the influence of liking and learning engagement from the perspectives of student–teacher and teacher–student. Based on the theoretical and existing findings, this study introduces psychological empowerment as a mediating variable and perceived teacher’s support as a moderating variable and proposes five hypotheses. The specific research mechanism is shown in Figure 1.

### Research samples and procedures

This study adopts questionnaire survey to obtain reliable and realistic first-hand research data. The data mainly come from vocational college students in China’s Pearl River Delta and Yangtze River Delta regions. Research data were distributed and collected by three people in a team, and the survey process was unified. The research investigator invited the administrators of each institution to assist in the questionnaire survey. The research investigator informed the management assistance of the institution about the details and research topics of the survey, answered the questions of the management assistance, and then asked the management assistance to invite their teachers to send the questionnaire survey to the students. To avoid the influence of common method deviation (Podsakoff et al., 2003), this study was divided into two-time nodes, with a 1-month interval, and the questionnaire lasted for 2 months (October–November 2021). At time node 1, students’ liking and perception of teacher’s support were investigated. At time node 2, the psychological empowerment and learning engagement of students were investigated. In addition, with the permission of the subjects, the research team added commonly used control variables, including gender, age, grade, education level, etc.

In this research, 1,800 questionnaires were collected for the first time, and 1,560 questionnaires were collected for the second time. After two time nodes of matching, the final valid matching questionnaires were 1,279. The effective rate of the questionnaire was 71.06%. In this questionnaire survey, there were 618 male students and 661 female students; there are 661 freshmen, 225 sophomores, and 393 juniors.

### Measurement of variables

To ensure the reliability and validity of the questionnaire, the existing mature scale was used for reference in this study. Before the survey, the scale was accurately translated into Chinese according to the standard translation and back-translation procedures (Brislin, 1986) and repeated proofreading with the questionnaire distribution team. The 5-point Likert scale was adopted for each study (1–5 in the students’ questionnaire indicated “strongly disagree” to “strongly agree”).

Liking: The 4-item scale compiled by Wayne and Ferris (1990) was used to measure students’ liking for teachers. Questions, for example, “I like my teaching teacher very much.” The Cronbach’s α coefficient of this scale in this study was 0.938.

Perceived teacher’s support: The questionnaire of students’ perceived teacher’s support prepared by Ou (2005) was used to investigate. This questionnaire involves three dimensions: learning support, emotional support, and ability support, with a total of 19 questions. Questions for example, “My teacher always supports me to participate in various activities and competitions.” The Cronbach’s α coefficient of this scale in this study was 0.962.

Psychological empowerment: The empowerment scale revised by Li-Chaoping and Shi-Kan (2006) according to the English version of Spreitzer (1995) was adopted. The questionnaire consists of 12 questions, which are divided into four dimensions: work meaning, self-determination, self-efficacy, and work influence. Questions for example, “The work I do is very meaningful to me.” The Cronbach’s α coefficient of this scale in this study was 0.933.

Learning engagement: Schaufeli et al. (2002) was used to compile the Student Learning Engagement Scale (UWES-S; Schaufeli et al., 2002), which involved three dimensions: vitality, dedication, and concentration, with a total of 17 questions. Questions like “When I study, I feel energetic.” The Cronbach’s α coefficient of this scale in this study was 0.976.

To prevent the influence of demographic variables on the results of the model, the gender, age, and grade of students were controlled in this study.

#### Analytical methods

In this study, SPSS22.0 and AMOS22.0 software were used for data analysis. Data analysis mainly included the common method deviation test, confirmatory factor analysis, correlation analysis, regression test, and bootstrap test. The specific data results are shown below.

## Data analysis and research results

### Common method deviation test

The questionnaire was completed and collected online. To reduce the deviation of the common method, the questionnaire was divided into two periods, but all the questionnaires were self-reported by the students. It is therefore necessary to have a common method bias for the data. Harman single factor test (Podsakoff et al., 2003) was adopted for factor analysis of all problems, and it was found that the explanatory variance of the first factor was 42.23%, less than the recommended value of 50%. Therefore, there is no significant bias, and the relationship between the variables is credible and does not affect the overall study results.

### Confirmatory factor analysis

To test the discriminant validity of the variables involved in this study, structural equation model was used to conduct factor analysis to test the variables and the model. As can be seen from Table 1, the four-factor model of the research hypothesis is better than other factor models in the fitting degree of sample data (χ^2^ = 5855.56, df = 1,228, RMSEA = 0.054, SRMR = 0.041, CFI = 0.938, TLI = 0.933), indicating that the questionnaire design of this study has good discriminating validity. It indicates that the questionnaire design in this study has good discriminant validity, and the four factors represent four different constructs, which can be used for regression analysis.

**TABLE 1:** Confirmatory factor analysis results (*N* = 1279).

Model	χ 2	df	*x*^2^/df	RMSEA	SRMR	CFI	TLI
Four factor model (hypothesis)	5855.56	1,228	4.768	0.054	0.041	0.938	0.933
Three factor model (A + B)	10919.13	1,231	8.870	0.078	0.112	0.870	0.859
Three factor model (A + C)	8267.54	1,231	6.716	0.067	0.046	0.905	0.898
Two factor model (A + B + C)	13137.33	1,233	10.655	0.087	0.200	0.840	0.828
Single factor model (A + B + C + D)	24408.79	1,234	19.780	0.121	0.203	0.688	0.665

A, like; B, psychological empowerment; C, perceived teacher’s support; D, learning engagement; “+”, indicates fusion.

### Correlation analysis

To further clarify the relationship between students’ liking, psychological empowerment, perceived teacher’s support, and learning engagement, this study conducted correlation analysis among variables. The results in Table 2 show that the correlation coefficients between variables in the hypothetical relationship are significant. This also provides support for the verification of the research hypothesis, but further verification is needed.

**TABLE 2:** Mean value, standard deviation, and correlation coefficient of variable (*N* = 1,279).

Variable	Mean value	Standard deviation	1	2	3	4	5	6
1. Gender	0.517	0.499						
2. Age	19.482	1.477	–0.028					
3. Grade	1.790	0.884	0.029	0.775**				
4. Liking	4.031	0.869	–0.104**	–0.038	–0.048			
5. Psychological empowerment	3.934	0.722	–0.203**	0.049	0.041	0.263**		
6. Perceived teacher’s support	3.769	0.709	–0.177**	–0.042	–0.037	0.696**	0.332**	
7. Learning engagement	3.859	0.739	–0.202**	0.043	0.036	0.246**	0.798**	0.340**

***p < 0.001, **p < 0.01, *p < 0.05 (the same below).

### Regression analysis

In this study, multiple linear regression and process methods were used to analyze the relationship among students’ liking, psychological empowerment, perceived teacher’s support, and learning engagement, with gender, age, and grade as the control variables. The results are shown in Table 3.

**TABLE 3:** Regression analysis model.

Variables	Psychological empowerment		Learning engagement	
	Model 1	Model 2	Model 3	Model 4
***Control variables***				
Gender	–0.257***	–0.136***	–0.178***	–0.040*
Age	0.010	0.029	0.015	–0.001
Grade	0.034	0.038	0.040	0.007
*Independent variables*				
Liking	0.206***	0.092*	0.230***	0.037*
*Mediating variable*				
Psychological empowerment				0.779***
***Moderating variables***				
Perceived teacher’s support		0.264***		
***Interactive items***				
Liking*Psychological empowerment		0.105***		
*R* ^2^	0.104	0.148	0.085	0.639
Δ*R*^2^	0.060	0.010	0.052	0.544*
*F*	36.910***	36.774***	33.293***	450.691***

***p < 0.001, **p < 0.01, *p < 0.05.

It can be seen from the results of model 3 in Table 3, after controlling for students’ natural attributes, students’ liking for teachers significantly positively affects learning engagement (β = 0.230, *p* < 0.001). Therefore, hypothesis 1 is true. It can be seen from the results of model 1 in Table 3 that, after controlling for students’ natural attributes, students’ liking for teachers has a significant positive impact on psychological empowerment (β = 0.206, *p* < 0.001), so hypothesis 2 is true. According to the results of model 4 in Table 3, after controlling for students’ natural attributes, psychological empowerment had a mediating effect on the relationship between liking and learning engagement (β = 0.779, *p* < 0.001). To further clarify this mediation effect, the bootstrap method was used for testing, and the results are shown in Table 4. After controlling for the students’ natural attributes, the indirect effect of psychological empowerment is significant and does not include 0 in the 95% confidence interval. Liking has a significant direct effect on learning engagement, and the confidence interval of 95% does not contain 0, indicating that anxiety plays a partial mediating role in the relationship between employees’ risk perception and job engagement. Therefore, hypothesis 3 is established.

**TABLE 4:** Bootstrap test of mediating effects.

	Effect size	Standard error	95% confidence interval	
			Lower limit	Upper limit
Indirect effects	0.032	0.015	0.002	0.061
Direct effects	0.164	0.024	0.119	0.215

Sample size of bootstrap *N* = 5,000.

**FIGURE 2:**
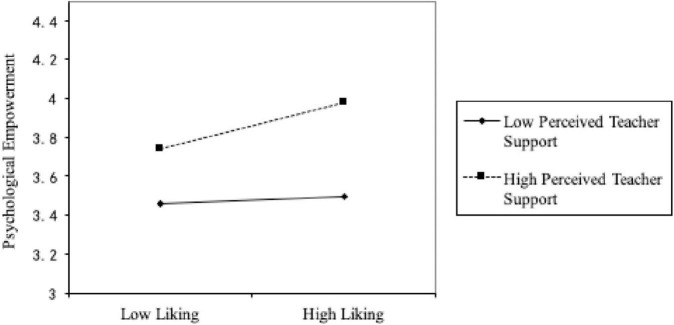
The effect of interaction between liking and perceived teacher support on psychological empowerment.

The moderating effect can be verified from the results of model 2 in Table 3 that after controlling for students’ natural attributes, the interaction between students’ liking and perceived teacher’s – support is significant (β = 0.105, *p* < 0.001). Therefore, perceived teacher’s support plays a moderating role in the relationship between student liking and psychological empowerment. To explain this significant regulatory effect, Aiken et al. (1991) were used to adjust the high and low levels of adjustment variables for one standard deviation (±1 SD) above or below the mean (plus or minus). The results are shown in Figure 2, which indicates that the higher the degree of students’ perceived teacher’s support, the stronger the positive relationship between students’ liking and psychological empowerment. Thus, hypothesis 4 is true.

To further test the moderated mediation, the bootstrap method was adopted to test the moderated mediation model based on the existing studies. The specific analysis results are shown in Table 5.

**TABLE 5:** Bootstrap test of moderated mediating effects.

Independent variable	Mediator variable	Moderator variable	Indirect effects	Standard error	95% confidence interval
Liking		Low(–SD)	0.015	0.030	[–0.041, 0.076]
	Psychological empowerment	Perceived teacher’s support			
		High (+ SD)	0.107	0.037	[0.040, 0.185]

Sample size of bootstrap *N* = 5,000.

With high level of perceived teacher’s support, the indirect effect value is 0.107, which is value at 95% confidence interval [0.040, 0.185]. It can be seen that perceived teacher’s support moderates the mediating role of psychological empowerment in the relationship between students’ liking and learning engagement. The mediating effect was stronger when the perceived teacher’s support was higher. So, hypothesis 5 is true.

## Conclusion

This study constructs a moderated mediation model based on social exchange theory. From the perspectives of students and teachers, this manuscript explores the positive mediating role of psychological empowerment in the relationship between students’ liking and learning engagement, and the positive moderating role of perceived teacher support in the relationship between students’ liking and psychological empowerment. The findings of the study have a certain theoretical and practical contribution to the improvement of students’ learning involvement, and also provide some help and guidance for vocational colleges to deal with the relationship between students and teachers.

## Discussion

### Theoretical significance

First, this study systematically identifies the causes of learning engagement. “Like,” as an active positive emotion, has important predictive significance for work and organization (Kong et al., 2017). From the perspective of students, liking teachers can positively affect students’ learning engagement, which makes a certain contribution to the research on the mechanism of learning engagement based on emotion-behavior theory.

Second, this study identifies the mediating role of psychological empowerment. This study found that psychological empowerment plays a partial mediating role between students’ liking and learning engagement and proved the mechanism of the effect of liking on students’ learning engagement. Students-like teachers will feel more psychological empowerment and show more commitment to learning. By revealing the mediating role of psychological empowerment in the relationship between students’ liking and learning engagement, it clarifies the action path of emotion-behavior: liking – psychological empowerment – learning engagement. It provides a new theoretical perspective for exploring the influence mechanism of liking and the reason mechanism of learning engagement and further enriched the empirical research literature on learning engagement.

Finally, this study found the moderating effect of perceived teacher’s support. This study explored the moderating effect of students’ perceived teacher’s support on the relationship among liking, psychological empowerment, and learning engagement and further deepened students’ understanding of the influencing process of learning engagement generated by liking teachers. Specifically, when students receive more support from teachers, such as resources, encouragement, and recognition from teachers, students will like teachers, and then, they will spend more energy and resources on study. Based on the social exchange theory, this study explores the boundary effect of teacher’s support on the research model from the student–teacher and teacher–student perspectives.

### Practical significance

First, these studies have confirmed that students’ liking for teachers has a positive impact on learning engagement (Dewaele and Li, 2021). First, students themselves should maintain a high degree of interest and liking for their teachers, because such positive emotions can help students to be more actively involved in learning. Second, teachers should cultivate students’ liking for teachers. When students have shown liking and admiration for teachers, teachers can keep students’ positive emotions by maintaining the teaching mode and communication mode. When students do not like teachers, teachers can change their own style to cultivate students’ interest in teaching and stimulate students’ liking emotions (Li et al., 2020). Third, teachers should improve the content of teaching courses according to the interests of students and cultivate students’ interest and favorable interest in the teacher (Milner, 2008). Finally, when recruiting teachers, vocational colleges should also consider the characteristics of teachers that students like (Carter Andrews et al., 2019).

Second, the study confirmed the mediating role of psychological empowerment between liking and learning engagement. When students like teachers, the level of psychological empowerment is higher, and psychological empowerment plays a bridge between liking and learning engagement. Like is a one-way emotion, while psychological empowerment is a comprehensive cognition and incentive that students really feel (Ahmed and Malik, 2019). It is very important to transform the one-way emotion of like into internal motivation to improve students’ learning engagement. Teachers should take the initiative to strengthen the communication with students and enhance the level of psychological empowerment of students, so that students’ liking plays a full role. Students should also take the initiative to get close to teachers, find and solve problems, maintain and improve the relationship with teachers, and enhance the level of psychological empowerment, so as to be more actively involved in learning (Muijs and Harris, 2006).

Finally, the study confirmed the moderating effect of perceived teacher’s support on the relationship among liking, psychological empowerment, and learning engagement. The higher the students’ perception of teacher’s support, the more resources and help they get from the teacher, and the more likely they are to be involved in learning. Teachers should provide students with more support and encouragement, so that students can increase their level of psychological authorization, work harder and more confident in learning, and actively engage in learning (Shen et al., 2009).

### Limitation and future research directions

This study explores the learning engagement of vocational college students. Although some valuable conclusions have been obtained, there are also the following limitations. First, although the survey data of multi-stage time points were adopted in this study, due to the large number of teachers and students, the support level of teachers for each student could not be accurately evaluated, so the single-source data of students’ self-reporting were adopted. Though this study also verified the bias through common methods, subsequent studies suggest a multi-source, multi-stage investigation approach. Second, this study is based on the level of students, neither consider the impact of students’ family atmosphere nor the impact of class learning atmosphere on their learning engagement. Future research suggests paying attention to whether the difference between learning team and class atmosphere has different effects on learning engagement. Finally, the data in this study are all from the Yangtze River Delta and Pearl River Delta in mainland China, and thus, the relationship between variables found in this study is universal for higher vocational colleges in other regions that need more research.

## Data availability statement

The raw data supporting the conclusions of this article will be made available by the authors, without undue reservation.

## Author contributions

LL designed the main idea and collected and analyzed the data. LW wrote the manuscript. LZ modified the research design. All authors contributed to the article and approved the submitted version.
